# Flavors of Uncertainty: The Difference between Denial and Debate

**DOI:** 10.1289/ehp.120-a314

**Published:** 2012-08-01

**Authors:** Wendee Holtcamp

**Affiliations:** **Wendee Holtcamp**, based in Houston, TX, has written for *Nature*, *Scientific American*, *National Wildlife*, and other magazines.


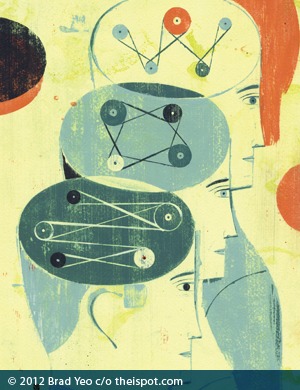
During the pilot episode of Comedy Central’s late night television show  *The Colbert Report*, satirist Stephen Colbert coined the term “truthiness”—truths that feel right regardless of evidence or reason.  Using sardonic wit he deadpanned, “Face it, folks: We are a divided nation. Not between Democrats and Republicans, or conservatives and liberals, or tops and bottoms. No. We are divided between those who think with their head and those who know with their heart.”^^1^^ Using satire, Colbert captured the essence of an issue that has many people deeply concerned: the denial of scientific evidence on  the basis of gut-level emotions.

Science denial sometimes occurs around environmental health issues. For example, some people catch and consume fish from polluted streams despite posted warnings. Some tan themselves without adequate protection against ultraviolet radiation. Others smoke cigarettes or live with secondhand smoke and believe they won’t succumb to illness. Still others burn garbage in barrels, ignoring laws and warnings regarding human health risks.

By many counts the level of science education and the general understanding of science in the United States, particularly relative to other nations, has stagnated or declined,^^2^^^^,^^^^3^^^^,^^^^4^^^^,^^^^5^^ and some denial results from a lack of knowledge about the scientific process. The public may not grasp the difference between the results of a single study, a handful of studies, and a scientific consensus, and such distinctions are not always communicated clearly by the media.

In other cases, industries and interest groups may drum up “organized doubt” in order to achieve a goal—for example, continued production and sale of a product, or advancement of a political agenda. Such campaigns have targeted the demonstrated health hazards of agents such as tobacco, lead, and DDT.^^6^^^^,^^^^7^^^^,^^^^8^^^^,^^^^9^^^^,^^^^10^^

The ensuing misinformation trickles down through the media to the public, resulting in confusion, exasperation, and distrust.^^5^^^^,^^^^11^^^^,^^^^12^^ “Science, for various reasons, has become more politicized,” says Terry Devitt, director of research communications at the University of Wisconsin–Madison. “Science, twenty years ago, used to have more cachet with the public, and that trust has been seriously eroded by coordinated attacks on science.” Devitt helped organize “Science Writing in the Age of Denial Conference,” one of the first conferences focusing exclusively on science denial, which was held at the university 23–24 April 2012.^^13^^

## Scientific or Cultural Controversy?

Distinguishing fact from spin and scientific debate from organized doubt is challenging in a rapidly changing media environment where, essentially, everyone has a printing press. If the public better understood how the media worked, it could help, says Gerald Markowitz, distinguished professor of history at the John Jay College of Criminal Justice at the City University of New York. “Most journalists and establishment media want to show both sides of an issue,” Markowitz says, “and so the fact that there’s controversy means they feel they’ve got to show what both sides are, whereas in fact, they’ve got to do a better job of investigating whether it’s a legitimate or a created controversy.”

In one of the keynote talks at the April conference, UW–Madison genetics and molecular biology professor Sean Carroll outlined what he calls “a general manual of denialism”—six tactics used time and again in denial campaigns since at least the nineteenth century.^^14^^^^,^^^^15^^ First, cast doubt on the science. Second, question the personal motives and integrity of the scientists. Third, magnify genuine disagreements among scientists, and cite nonexperts with minority opinions as authorities. Fourth, exaggerate the potential harm caused by the issue at hand. Fifth, frame issues as a threat to personal freedom. And sixth, claim that acceptance would repudiate a key philosophy, religious belief, or practice of a group. Carroll says this blueprint can help people distinguish denial from legitimate scientific debate on various issues.

But while it may be relatively easy to spot some of these tactics, others can be more challenging to detect. If, as research suggests, people get their information about science topics largely from television and the Internet,^^11^^ and if media outlets are not clarifying the differences between individual studies and scientific consensus views, then the public may face serious challenges in distinguishing fact from spin.

Additionally, there is a difference between legitimate scientific debate or uncertainty, and a cultural, political or religious controversy over a scientific issue. But if reporters do not explain the distinction, this, too, can skew the public’s understanding of science. “Climate change, stem cells, synthetic biology—these are issues where every survey shows the public trusts scientists to do the science right,”^^5^^^^,^^^^16^^^^,^^^^17^^ says Dietram Scheufele, the John E. Ross chair in science communication at UW–Madison and a panelist at the April conference. “The key questions the public is concerned with are not the scientific aspects, but the ethical, legal, and social implications.”

“Knowledge alone does not yield appropriate action.”

—Naomi Oreskes, University of California, San Diego

“Any theory of motivated reasoning has to capture the nuance that what we believe is some compromise between what we want to believe and what [our survival] will let us believe. . . .  With clear enough information, people believe things even when they don’t want to believe them.”

—Peter Ditto, University of California, Irvine

“Most journalists and establishment media want to show both sides of an issue, and so the fact that there’s controversy means they feel they’ve got to show what both sides are, whereas in fact, they’ve got to do a better job of investigating whether it’s a legitimate or a created controversy.”

—Gerald Markowitz, City University of New York

“Climate change, stem cells, synthetic biology—these are issues where every survey shows the public trusts scientists to do the science right. The key questions the public is concerned with are not the scientific aspects, but the ethical, legal, and social implications.”

—Dietram Scheufele, University of Wisconsin–Madison

University of Michigan political science professor Arthur Lupia, who also spoke at the April conference, agrees. “When we’re fundamentally getting down to questions of what society should do, science can inform that debate, but ultimately you need a moral or ethical basis to make decisions,” he says.

“Corporations and industry groups have been very effective at hiding their affiliations and raising skepticism,” says Markowitz. “They just have to play on the good part of science, which is that science is skeptical. But when that skepticism is used for a purpose of protecting an industry, then it’s perverting what science is [about].”

For example, Markowitz and colleague David Rosner are publishing a book in spring 2013 about the lead industry’s organized doubt campaign. For decades, the lead industry denied a growing body of research on the toxicity of lead. As a result, Markowitz says, “You had a large number of doctors who didn’t test children for lead, and parents were not aware of the dangers that their children faced from tiny amounts of lead.”

This organized doubt trickles down to the voting public, who may not recognize a misinformation campaign for what it is, according to Markowitz. “Corporate advertising plays very much [into] this idea that we’re all responsible for our own lives, and we don’t want the government to tell us what to do. That is a very powerful message because individualism is very much a part of U.S. identity,” he says. Yet it often goes against our own best interest, he says: “It’s not in anybody’s self-interest to be poisoned by the air we breathe. That’s not freedom. That’s insanity.” But as long as there appears to be reasonable doubt of a particular scientific finding, he says, individuals seem more inclined to ignore evidence, often at their own peril.

## Conveying the Message Correctly

“I’m here with the very depressing conclusion that knowledge isn’t power,” said Naomi Oreskes, a professor of history and science studies at the University of California, San Diego, during her keynote address at the April conference.^^18^^ “If people don’t like the implications of your knowledge, they will resist, reject, and even attack it,” she says today. “Knowledge alone does not yield appropriate action.” But how do scientists and journalists educate a public who may be denying a particular scientific consensus?

At the conference Lupia told the audience, “We get the idea that if we just tell [people] what we know, they will change how they think and what they do.”^^19^^ As scientists and science communicators, we have something of value to share, he explained, and we expect our message to come across like a shiny new sports car wrapped in a bow—but the reality is often more like an old rust bucket rolling into a lake.

The problem, Lupia said, is our expectations of the audience. He offered an analogy: Let’s say you are an expert in a particular patch of woods. A friend strolling through the woods gets lost. If you first determine where the friend is, you could give him detailed directions for how to navigate the woods. But yelling directions without any knowledge of where the friend is just gets him more lost.

Whether giving a keynote address or posting on Facebook, people can merely affirm their own values and blame listeners when they don’t agree, or they can try to genuinely connect with people. Atmospheric scientist Katharine Hayhoe dealt with this issue when she accepted a position at Texas Tech University, which is located in socially and politically conservative West Texas. Hayhoe was welcomed more warmly by the Lubbock social scene than she expected, possibly because she shared the city’s predominant religious affiliation. Women’s groups, churches, and grade schools invited her to speak, and she believes the close interactions positively influenced how people received what she had to say about climate change.

Hayhoe says that political conservatives have repeatedly told her they feel pigeonholed as stupid if they disagree with the science, yet they have good questions that Hayhoe feels deserve answers rather than dismissals. That’s why she and her husband wrote *A Climate for Change: Global Warming Facts for Faith-Based Decisions*. Her husband, the pastor of a nondenominational evangelical church, was a climate change denier when they married. As he examined the evidence, she says he began to accept the consensus view that emissions from human activities are a primary factor in the Earth’s temperature rise.

One faith-based approach to discussing climate change and other environmental health topics is “creation care,” the idea of restoring humans to their rightful role as stewards of creation.^^20^^ The creation care/stewardship message has resonated with the leadership of many Christian denominations as well as other religions. However, one size does not fit all when it comes to communicating about science, and Hayhoe cautions that conservative Christians may hear a different message in the concept of creation care.

“Leading with a discussion about caring for creation feeds directly into the misconception that if there is a conflict between people and the environment, environmentalists would rather throw the people under the bus,” she says. “Also, the message of stewardship is one of failure: ‘We had a responsibility from God, and we have failed, we have sinned, we are bad people.’ Motivation for change? I say no.” Hayhoe believes that “heaping on the guilt is no driver of permanent, long-term change for the average person.” Similarly, Oreskes said at the conference, dire warnings that not acting on climate change means killing the citizens of the Maldives can overwhelm people and turn them off rather than incite them to act.

What is the alternative? First, Hayhoe suggests, offer a positive message about what we can do right as opposed to a negative message about all the things we’ve done wrong. Second, refrain from alarmism. And third, give concrete examples of ways to take action. Hayhoe believes imparting a call to action with concrete examples of what a concerned citizen can do motivates change far more effectively than guilt-inducing messages. The same applies to other issues affected by denialism, she says: It’s better to connect than to dismiss, and to encourage than to shame.

## Motivated Reasoning

Hayhoe’s ideas align with research on motivated reasoning, or the factors and goals that underlie people’s thinking processes. When people are deciding what messages they want to pay attention to, they aren’t always driven by a desire to find the most factually accurate answer; instead, Lupia says, “A lot rides on protection of self esteem.”^^21^^^^,^^^^22^^

At the April conference Lupia noted that “for contested issues, high credibility is a must.” Furthermore, he said, repeatedly hearing the same message from the same expected sources doesn’t make a difference in terms of the message getting through, but hearing it from an unexpected source—for example, someone of a faith or political background not typically aligned with that message—does.

On the other hand, “beliefs, even very implausible ones, can be held in place through social support,”^^23^^^^,^^^^24^^ says Peter Ditto, a professor of psychology and social behavior at the University of California, Irvine. “If I hear that global warming science is all just made up . . . and that sounds not quite right to me, but then I tune in to [media outlets where] I hear lots of other people who all seem pretty credible who believe this too, that initially implausible-sounding idea starts to sound much more plausible.”

Scheufele explains there is a science behind heuristics, or the tools people use to simplify, understand, and integrate complex information into their worldview. His research supports the psychological theory that people are “cognitive misers” who typically base their opinions and decisions on the least amount of information possible (or the most easily accessible information) rather than reading original research or legislation.^^25^^ In psychology circles, the cognitive miser model contrasts with the “scientific literacy model,” which posits that if people just knew the science, they would support its progress and implications.

The two models may, ultimately, be complementary.^^26^^ Scheuefele has studied how laypeople and scientists develop opinions, using nanotechnology as a focus.^^27^^ “Scientists use the same heuristics that the lay public uses,” he says. “Professional judgments mean a lot, but so does ideology.”

When it comes to matters involving one’s own well-being—be it a personal health scare or the implications of climate change—people don’t automatically turn away from scary information, at least initially, according to Ditto. “They turn toward it and see if they can ‘think it away,’ ” he says. “Because they are more skeptical about it, it takes more information to make them believe something they don’t want to believe.”^^28^^^^,^^^^29^^^^,^^^^30^^ As he puts it, one doctor can convince you that you are healthy, but you want a second opinion when a doctor says you have a terminal illness.

“Any theory of motivated reasoning has to capture the nuance that what we believe is some compromise between what we want to believe and what [our survival] will let us believe,”^^29^^^^,^^^^31^^ Ditto said at the April conference. “Believing things are a bit more positive than they are may be helpful, but if those positive illusions get too far out of alignment with reality, then things can get problematic,” he says today. “My research suggests that with clear enough information, people believe things even when they don’t want to believe them. Everyone will accept the validity of climate science once they’re ankle-deep in ocean water.”
